# Incorporation of Metal-Based Nanoadditives into the PLA Matrix: Effect of Surface Properties on Antibacterial Activity and Mechanical Performance of PLA Nanoadditive Films

**DOI:** 10.3390/molecules26144161

**Published:** 2021-07-08

**Authors:** Klementina Pušnik Črešnar, Alexandra Aulova, Dimitrios N. Bikiaris, Dimitra Lambropoulou, Katja Kuzmič, Lidija Fras Zemljič

**Affiliations:** 1 Faculty of Mechanical Engineering, University of Maribor, 2000 Maribor, Slovenia; katja.kuzmic@student.um.si; 2 Faculty of Mechanical Engineering, University of Ljubljana, 1000 Ljubljana, Slovenia; alexandra.aulova@fs.uni-lj.si; 3 Laboratory of Polymer Chemistry and Technology, Department of Chemistry, Aristotle University of Thessaloniki, GR-541 24 Thessaloniki, Greece; dbic@chem.auth.gr; 4 Laboratory of Environmental Pollution Control, Department of Chemistry, Aristotle University of Thessaloniki, GR-541 24 Thessaloniki, Greece; dlambro@chem.auth.gr; 5 Centre for Interdisciplinary Research and Innovation (CIRI-AUTH), Balkan Center, 10th km Thessaloniki-Thermi Rd, GR 57001 Thessaloniki, Greece

**Keywords:** poly(lactic acid), nanoparticles, composite additive films, SEM analysis, surface free energy calculation, antibacterial activity, nanoindentation

## Abstract

In this work, the modification process of poly(lactic acid) (PLA) with metal-based nanoparticle (NPs) additives (Ag, ZnO, TiO_2_) at different loading (0.5, 1.0, and 2.5 wt%) and by melt-mix extrusion method followed by film formation as one of the advantageous techniques for industrial application have been investigated. PLA nanoparticle composite films (PLA-NPs) of PLA-Ag, PLA-ZnO, PLA-TiO_2_ were fabricated, allowing convenient dispersion of NPs within the PLA matrix to further pursue the challenge of investigating the surface properties of PLA-NPs reinforced plastics (as films) for the final functional properties, such as antimicrobial activity and surface mechanical properties. The main objective was to clarify how the addition of NPs to the PLA during the melt extrusion process affects the chemistry, morphology, and wettability of the surface and its further influence on the antibacterial efficiency and mechanical properties of the PLA-NPs. Therefore, the effect of Ag, ZnO, and TiO_2_ NPs incorporation on the morphology (SEM), elemental mapping analysis (SEM-EDX), roughness, surface free energy (SFE) of PLA-NPs measured by goniometry and calculated by OWRK (Owens, Wendt, Rabel, and Kaelble) model was evaluated and correlated with the final functional properties such as antimicrobial activity and surface mechanical properties. The developed PLA-metal-based nanocomposites, with improved mechanical and antimicrobial surface properties, could be used as sustainable and biodegradable materials, offering desirable multifunctionalities not only for food packaging but also for cosmetics and hygiene products, as well as for broader plastic products where antimicrobial activity is desirable.

## 1. Introduction

The development of plastic packaging materials is an extremely interesting area of research that has shown rapid growth in recent years. The focus is on the improvement of materials, which, on the one hand, protect the product and extend its life, and on the other hand, have a biodegradable and functional aspect. In this regard, petroleum-based plastics such as polyethylene (PE), polypropylene (PP), polyamide (PA) have experienced exponential growth every year due to their easy availability, low cost, good barrier, and mechanical properties for packaging. Despite their good properties, the tremendous growth and accumulation of these types of plastics in the world is forcing the plastics industry to pay close attention to them. Thus, research efforts are even being made to develop sustainable and functional packaging material [1,2,3,4].

In general, packaging novelty concepts have focused not only on biodegradability but also on multifunctionality, in addition to the creation of bio-based and/or biodegradable plastics using biopolymers such as poly(lactic acid) PLA. In particular, surface coating strategies or the integration of nanoparticles (NPs) additives into polymer matrices are used [5,6,7,8,9]. Similar to bulk additives, NPs additives are incorporated into the polymer matrix, but on the other hand, the aspect ratio of largest to smallest dimension provides increased reinforcement effect in the preparation of nanocomposites-based PLA. Moreover, this technology shows some advantages compared to coatings, while beside the improvement of mechanical properties, the introduction of stability of NPs-additives incorporation without possible migration in media, which is especially appreciated in packaging. The clay-based nanoadditives [10,11,12,13,14], nanocellulose [15,16,17,18,19,20,21,22], carbon nanotubes [23,24,25], silica NPs [26], metal and metal oxide NPs [27,28,29,30,31,32,33,34,35,36] which, within the PLA matrix, represent one of the widely studied solutions for the preparation of multifunctional PLA nanocomposites, improving physicochemical, mechanical properties as well as providing thermal and additional antibacterial efficacy and protection of the [37,38,39,40,41,42]. In general, the modification of PLA-based NPs composites (PLA-NPs) represents a key strategy for the advantage of maintenance-free sterile surfaces, which are aimed at many plastic products [39]. Several studies have been highlighted of PLA metal-based NPs composites films; silver (Ag) NPs composites, zinc oxide (ZnO) NPs composites, and titanium dioxide (TiO_2_) NPs composite films prepared by methods such as in-situ polymerization, solvent casting, electrospinning, extrusion process, which improved good thermal properties and noticeable tensile strength depending on the surface modification of the fillers [34,43,44,45,46] and exceeded antibacterial properties [47,48,49]. Although the additions of, i.e., Ag, ZnO, TiO_2,_ NPs have an important effect on the cold crystallization kinetics, thermal degradation, semi-crystalline morphology, and segmental dynamics of PLA-NPs composites studied by our previous research [36,50], the exact mechanism for their involvement and the distribution in the surface and consequently their dependency on surface properties of PLA-NPs composites is not fully understood. Nevertheless, it is the surface or the outermost layer of atoms/molecules of solid that really outlines how the material interacts with its environment and how it behaves for its envisioned purpose. These surface properties affect processes such as wetting, diffusion, adsorption (desorption) of macro/molecules as well as biomedia (microorganisms), and in this way controls interaction phenomena after the two materials come into contact and states the material functionality. The latter is of great importance in packaging concepts where the material acts at the interface with packaged media and thus surface functionality is the driving force for their efficiency. Therefore, understanding surface phenomena is crucial, and conversely, a deeper understanding of surface parameters’ influence on final functionality can enable its reverse manipulation for material optimization.

However, when NPs are integrated into the PLA polymer matrix, the surface properties of the PLA-NPs depend on the quality and quantity of NPs in the PLA matrix and the strength of adhesion at the PLA-NPs interface. In addition, the fabrication and performance of PLA-NPs are affected by surface forces that influence the dispersion between NPs and thermoplastic biopolymers such as PLA. The dispersion of NPs in the PLA matrix is closely related to the balance between the cohesive forces between NPs and biopolymers, which determine the extent of dispersion that can be achieved [51,52]. Contrary, the mechanical properties and thermodynamic stability of PLA-NPs are related to the strength of PLA-NPs interactions. The latter can be tuned by modifying the polymer or the NPs [6,44,53,54,55,56,57,58,59,60,61,62].

In this paper, we introduced the modification process of PLA with metal and metal oxide NPs by the melt-mix extrusion method followed by film formation as one of the advantageous techniques for industrial application. PLA-NPs composite films based on silver (PLA-Ag), zinc oxide (PLA-ZnO), and TiO_2_ (PLA-TiO_2_) were fabricated toward the convenient dispersion of the NPs within the PLA matrix to further address the challenge of studying the surface properties of the PLA-NPs reinforced plastics (as films) on final functional properties, such as antimicrobial activity and surface mechanical properties. The main research aim was to clarify how the addition of NPs to the PLA during the extrusion process affects the chemistry, morphology, and wettability of the surface of PLA-NPs composites film and their further influence on antibacterial as well as surface mechanical properties. Therefore, the effect of Ag, ZnO, and TiO_2_ NPs incorporation on the morphology (SEM), elemental mapping analysis and roughness as well as the surface free energy of PLA-NPs composites films, measured by goniometry and calculated by the WORK (Owens, Wendt, Rabel, and Kaelble) model, was evaluated and correlated to final, functional properties as antimicrobial activity and surface mechanical properties. To the best of our knowledge, such a study has not been presented before and thus represents the novelty in this research.

Such engineered PLA-NPs with improved antimicrobial and mechanical surface properties could be used as sustainable and biodegradable materials and provide desirable multifunctionalities not only in food packaging but also for cosmetics and hygiene products for consumers as well as for wider plastics products where antimicrobial activity is desired (organic waste bins, waste container).

**Figure 1:**
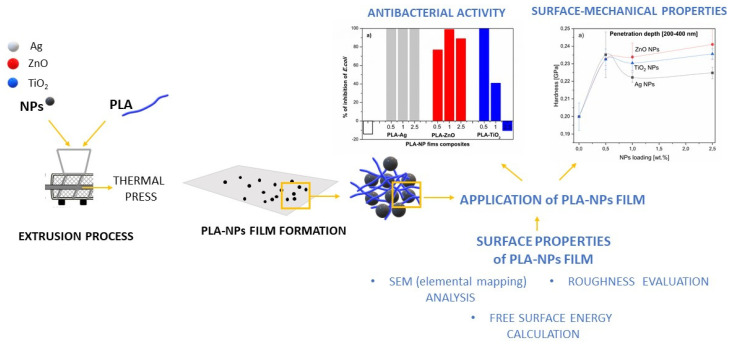
Schematic of PLA-based NPs film preparation and study.

A schematic presentation of our work is given in Figure 1.

## 2. Results

### 2.1. Properties of NPs

**Figure 2:**
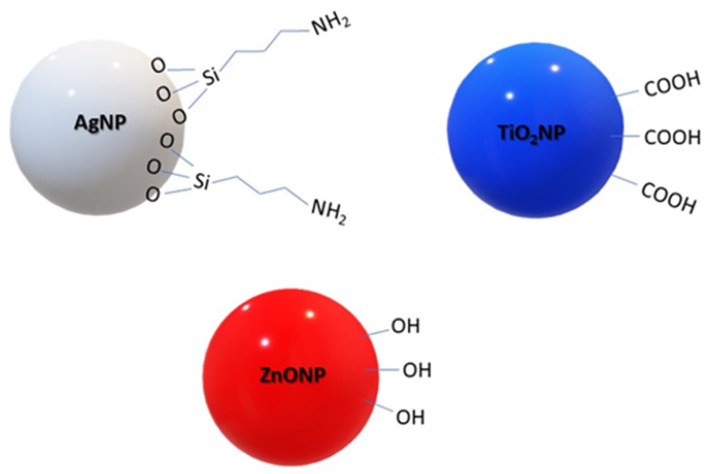
Scheme of the Ag, ZnO, and TiO_2_ NP.

The presence of metal-based NPs as a functional additive can change the surface properties of PLA-NPs composite film. In order to present the influence of NPs onto the surface properties of PLA-NPs composite films, the characterization of NPs using XRD measurements, TEM analysis, and ATR-FTIR spectroscopy, and zeta potential measurements is first presented. NPs used in this research have the following structure as shown in Figure 2.

**Figure 3:**
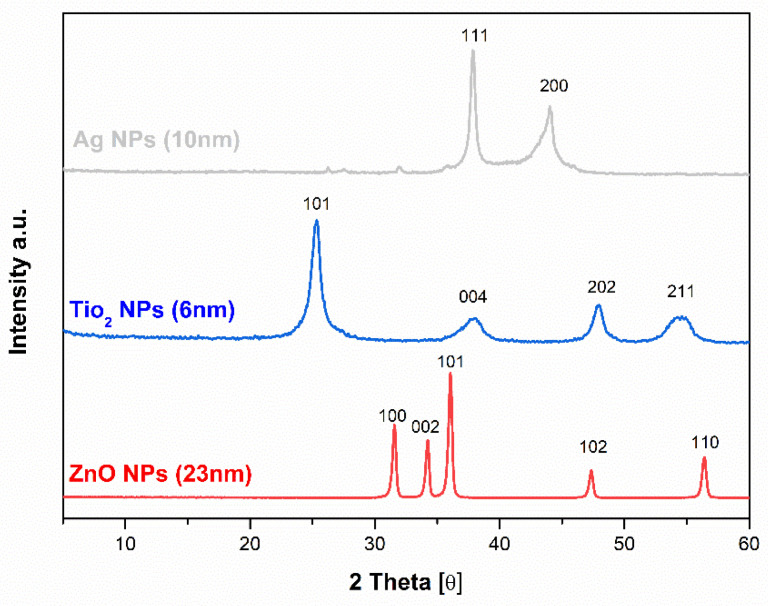
X-ray diffraction patterns of Ag NPs, TiO_2_ NPs, and ZnO NPs with a calculated average size of crystallite.

In Figure 3 the comparison of X-ray powder diffractograms of Ag, ZnO, and TiO_2_ NPs were evaluated. The XRD patterns of Ag NPs at 2θ of 37.84, 43.74 could explain the (111) and (200) crystallographic planes of the face-centered cubic Ag crystals. From the broadening of the XRD reflections the average crystallite size calculated from the Scherrer equation was estimated to be 10 nm, respectively [63,64]. The patterns of the next used NPs in PLA-NPs film composites TiO_2_ showed intense peaks that correspond to the typical signals of TiO_2_ are attributed to the anatase. The TiO_2_ structure with (101), (004), (202), and (211) crystal reflection planes that are aligned to the diffraction peaks at 2θ of 25.28, 37.80, 47.89, 54.41, and 6 nm average crystallite size were determined [65]. The patterns of third used NPs in PLA composites ZnO showed very sharp peaks corresponding to crystal planes (100), (002), (101), (102), and (110). These XRD patterns could be attributed to the formation wurtzite phase structure with a calculated (Scherrer equation) average crystallite size of 23 nm [66,67].

**Figure 4:**
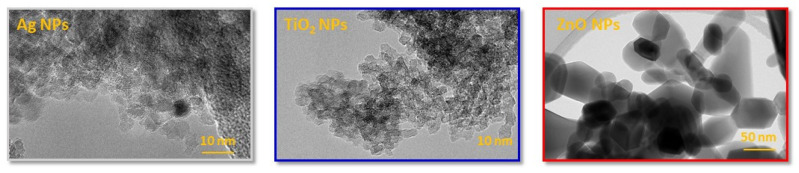
TEM images of metal-based NPs; Ag NPs, TiO_2_ NPs, and ZnO NPs.

The shape and morphology of the NPs measured with TEM analysis are mainly classified according to their dimensions. The TEM images represented in Figure 4 reveal the shape of NPs which include: one dimensional (1D) nanofillers that are usually in the form of nanoplatellets like ZnO NPs and three dimensional (3D) equiaxed nanoparticles in the form of spherical shapes like Ag NPs and TiO_2_ NPs (Figure 4) [52]. From Figure 4, it can be seen that Ag NPs and TiO_2_ NPs exhibited a spherical shape. On the other hand in the TEM images of the ZnO NPs appear as larger particles in comparison to Ag and TiO_2_ with both a spherical and hexagonal shape of NPs.

**Figure 5:**
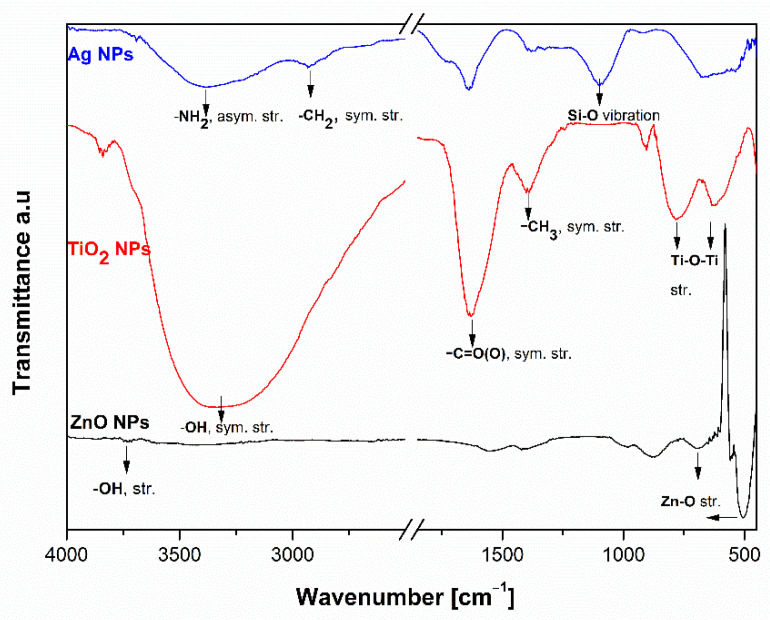
ATR-FTIR spectra of ZnO, Ag, and TiO_2_ NPs.

NPs were also characterized by ATR-FTIR spectroscopy. The ATR-FTIR spectra of Ag NPs, TiO_2_ NPs, and ZnO NPs are shown in Figure 5. The characteristic vibration peaks of Ag NPs are seen in the range of 4000 cm^−1^ to 500 cm^−1^ (Figure 5). The broad absorption peak at 3398 cm^−1^ shows the O-H stretching modes or the bending of the O-H group and indicates the presence of water molecules or hydroxyl groups adsorbed on the surface and the asymmetric NH_2_ stretching vibration. The presence of symmetric CH_2_ stretching vibrations is at about 2900 and 2850 cm^−1^ and indicates the presence of aliphatic chains. The absorption peak at 1630 cm^−1^ is attributed to adsorbed water on Ag NPs. The siloxane Si-O group stretching vibration peaks are characterized by strong characteristic peak vibrations at 1100 cm^−1^, which include Si-O stretching. Generally, the ATR-FTIR study of Ag NPs was carried out in the wavelength range of 4000–600 cm^−1^ to determine the adsorbed APS (aminopropylsilane) molecules onto the Ag NPs [68,69]. In contrast to Ag NPs, the ATR-FTIR spectra of TiO_2_ NPs in the range of (4000 to 600) cm^−1^ show the presence of carboxyl groups adsorbed on the surfaces of TiO_2_ NPs groups and surface adsorbed water molecules. In fact, the characteristic peak vibration at about 1640 cm^−1^ is assigned to the symmetric stretching of carboxyl groups (^−^C=O-O) and the peak at 1398 cm^−1^ can be attributed to the symmetric stretching of the CH_3_ group. The typical ATR-FTIR characteristic vibration occurring in the region below 800 cm^−1^ is pronounced with the metal-oxide, Ti-O-Ti vibrational mode. The ATR-FTIR spectra of ZnO NPs are also shown in Figure 5. The typical characteristic peak around 3704 cm^−1^ was observed, which corresponds to the stretching vibration of the isolated hydroxyl group bound to the metal center. The characteristic peaks of ZnO vibration in the region below 800 cm^−1^ and between 421 and 560 cm^−1^ show the presence of Zn-O stretching vibration, thus confirming the formation of ZnO [70,71,72,73].

The presented ATR-FTIR spectra of Ag NPs, TiO_2_ NPs, and ZnO NPs indicate, in all cases, the presence of the typical elemental composition of the NPs as well as the functional groups stabilizing the NPs (see Figure 1). The evaluated characteristic peaks vibration indicates the presence of aminopropylsilane adsorbed on the Ag NPs on the one hand and the carboxyl group on the surface of TiO_2_ NPs. By ZnO NPs, OH groups are residues of co-precipitation reactants oriented to adsorb on the surface. The ATR-FTIR spectra of ZnO NPs indicate these molecules on the surface as O-H stretching, shown in Figure 5.

**Figure 6:**
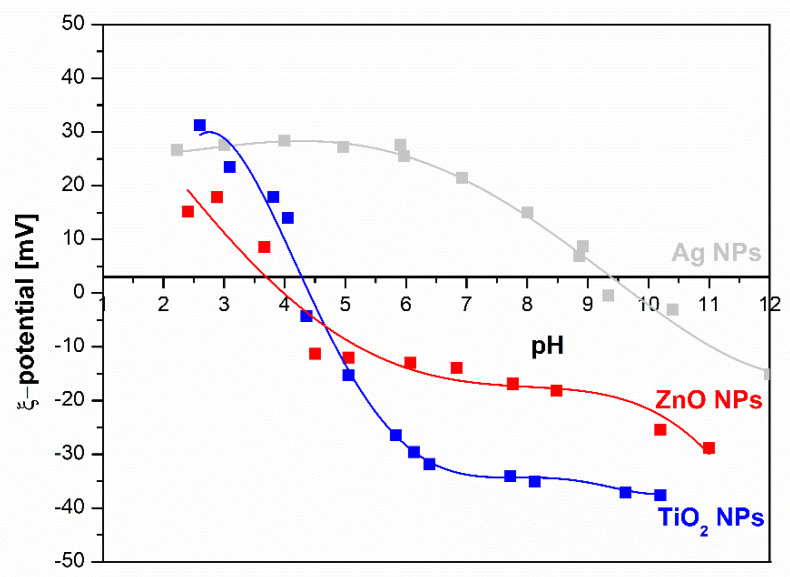
Zeta potential of water dispersion of Ag NPs, TiO_2_ NPs, and ZnO NPs as a function of pH.

Zeta potential measurements of NPs dispersion (Ag NPs, TiO_2_ NPs, and ZnO NPs) as a function of pH indicate of NPs surface charge are represented in Figure 6.

The Ag NPs dispersions exhibited an isoelectric point (IEP) close to 10, indicating the presence of positively charged groups on the surface of Ag NPs. In Figure 2. it is clear that amino groups should be present on the outer surface of the particles, which was proved by the zeta potential measurement and the ATR-FTIR measurements. The latter is also evidenced by the positive plateau value of the zeta potential. For the other two dispersions of TiO_2_ NPs and ZnO NPs, the IEP of 4.5 for TiO_2_ NPs and 4 for ZnO NPs, respectively, shows an acidic character, as also evidenced by the negative plateau zeta potential value due to the ionization of carboxyl groups (deprotonation) and OH groups (specific ions adsorption).

These metal-based NPs were selected as an additive and added to the PLA to achieve the functional properties of the final PLA-NPs film composites in conjunction with improved antibacterial properties and mechanical performance which is very much appreciated by plastic products, especially in the packaging segment. To gain more insight into the surface chemistry of the PLA composite films, additional SEM analysis (morphology), roughness measurements, goniometry with the calculation of SFE were chosen as techniques to understand the influence of surface properties onto antibacterial and mechanical properties.

### 2.2. Properties of PLA-NPS Film Composite

#### 2.2.1. Morphology of PLA-NPs Films Composite

**Figure 7:**
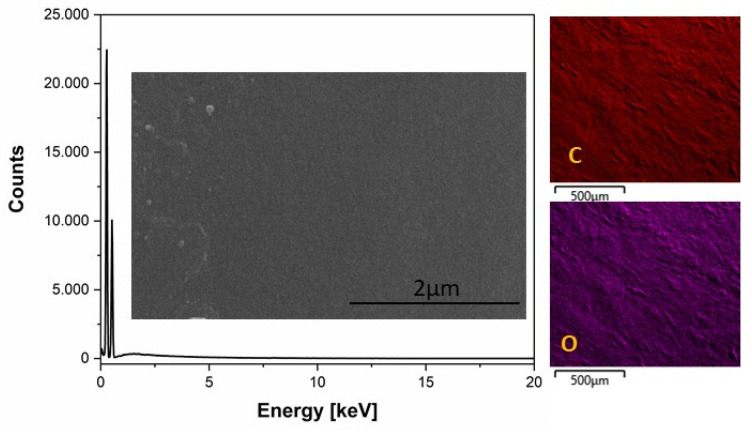
EDX spectrum of neat PLA with inset of SEM images of PLA and elemental mapping images of C and O.

SEM analysis was performed to investigate the surface morphology of neat PLA film, PLA-NPs composites films and distribution of NPs within PLA film matrix. The first part of the evaluation of the PLA-NPs film composites was focusing on the study of PLA-NPs composites film evaluated with EDX mapping analysis. The surface morphology of the neat PLA film is shown in insets Figure 7. The high magnification images of neat PLA represent a structure with smooth and flat appearance surfaces (Figure 7). The insets with EDX mapping images in Figure 6 represented the position of elements in the PLA matrix.

**Figure 8:**
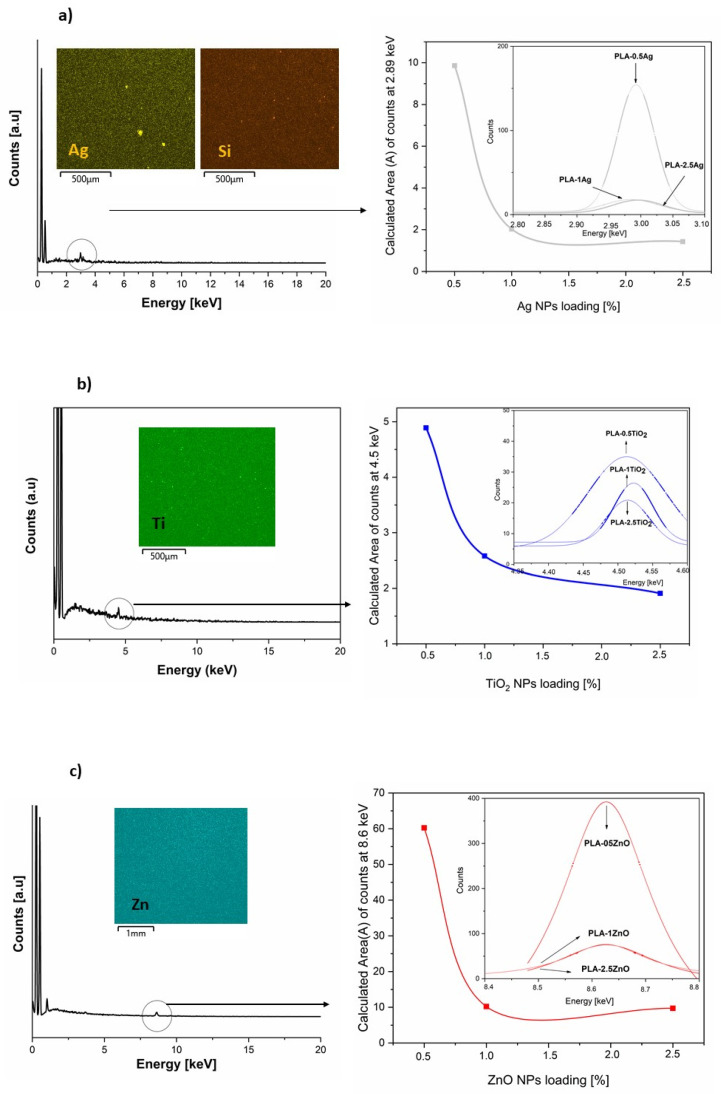
The EDX spectrum of PLA-NPs composite films with elemental mapping images of (**a**) PLA-Ag, (**b**) PLA-TiO_2_, and (**c**) PLA-ZnO were shown with the actual position of (**a**) Ag (located at 2.9 keV) and Si (at 1.7 keV), (**b**) Ti (located at 4.5 keV), and (**c**) Zn (located at 8.6 keV). The insets of the calculated area of all three PLA-NPs were compared as a function of area and NPs loading were represented.

In order to confirm the incorporation of NPs in the PLA-NPs modified films, the SEM-EDX spectrum mapping analysis of the neat PLA and the PLA loaded with all three different NPs concentrations: (i) 0.5%, (ii) 1%, and (iii) 2.5% were additionally presented and examined with their approximate atomic compositions. The first of the PLA-NPs composite films studied was based on PLA-Ag. The insets in Figure 8a showed the actual position of the represented elements for EDX analysis in the PLA-Ag film composites and confirmed the presence of Ag in PLA accordingly. The distinct counts of the PLA-Ag film reached the peak at 2.89keV, while this was not the case for the neat PLA, proving the incorporation of Ag NPs into the PLA film. Indeed, the insets showed that Si atoms were also detected by EDX mapping analysis, indicating the presence of main Si atoms of the APS molecules stabilized on the surfaces of the Ag NPs, which was already explained in our previous part, the NPs ATR-FTIR characterization. Further EDX mapping analysis, shown in Figure 8, revealed that as the NPs content in the PLA matrix increased, the Ag NPs whose Ag element was detected at 2.89 keV decreased in the PLA matrix. To find out approximately the preferred distribution of NPs in PLA-NPs film composites at different layers of PLA-Ag composite, the calculation of the average area of counts at 2.89 keV for Ag elements was also critically analyzed by fitting mathematical models (Gaussian terms) and calculation of an average area (A). The results for A (area) were plotted as a function of NPs loading in PLA-NPs film composites. The insets in Figure 8 showed the dependence of the area of Ag represented counts on NPs loading. Area of count for Ag NPs decreased with increasing NPs loading in PLA-NPs film composites.

The SEM EDX spectrum mapping analysis of PLA loaded TiO_2_ NPs (0.5%, 1.0%, and 2.5% NPs) is shown in Figure 8b. The EDX spectrum of all loaded NPs in PLA-TiO_2_ film composites confirmed the existence of Ti in PLA matrix composites, with the representative values of PLA-NPs film each reaching the peak at 4.5 keV. To find out more details about the distribution of TiO_2_ NPs in the final PLA film composites, the comparison of an average range of counts presented at 4.5 keV was also determined for Ti elements. As in the previous PLA-NPs composites based on Ag NPs, the results for A (area) were also plotted as a function of NPs loading in these PLA-NPs films based on TiO_2_ NPs. The insets in Figure 8b showed the calculation of the average area of the distinct counts for TiO_2_ NPs at 4.5 keV. The results showed that the calculated area for Ti decreased with increasing NPs loading in PLA-NPs film composites.

The last NPs used in PLA-NPs film composites was ZnO. The current position of the elements in the PLA-ZnO film is shown in Figure 8c, which was measured by EDX analysis. The indicated counts of PLA-ZnO peak at 8.6 keV, which proved that the Zn was contained in the PLA-ZnO film composites were measured. The comparison of elemental mapping analysis of all loaded PLA-ZnO film composites showed an improved distribution of ZnO NPs in the PLA film. Moreover, as the mass fraction of ZnO NPs increases, the EDX mapping analysis shows a lower distribution of NPs in PLA matrix. The dependence of ZnO loading on the average count at 8.6 keV was evaluated as in the previous two PLA-NPs film composites. The insets in Figure 8c show the dependence of the area of the counts known for ZnO on NPs loading. The area of counts for ZnO NPs decreased with increasing NPs loading in PLA-NPs film composites.

**Figure 9:**
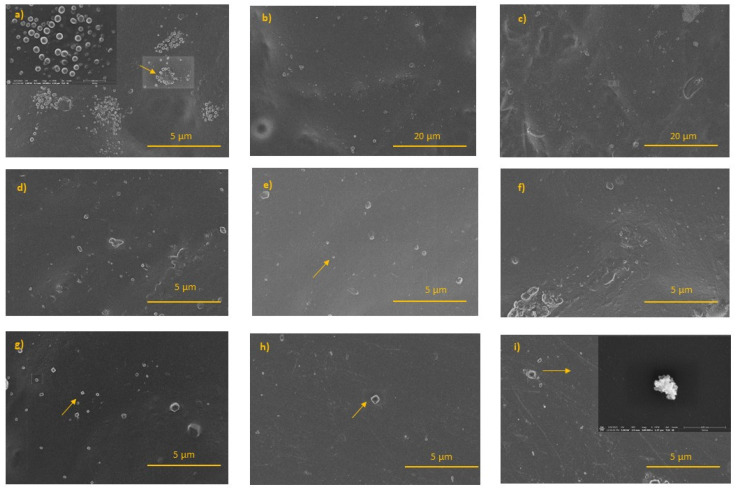
SEM images of the PLA-NPs film composites: (**a**) PLA-0.5Ag, (**b**) PLA-1.0Ag, (**c**) PLA-2.5Ag, (**d**) PLA-0.5TiO_2_, (**e**) PLA-1.0TiO_2_, (**f**) PLA-2.5TiO_2_, (**g**) PLA-0.5ZnO, (**h**) PLA-1.0ZnO, and (**i**) PLA-2.5ZnO.

The SEM images of PLA-NPs film composites were studied in Figure 9. PLA-Ag film composites shown in Figure 9a,b,c show spherical Ag NPs covering the PLA surfaces, with a size of about 80–120 nm of Ag NPs. The previous results of XRD and TEM analysis show that the Ag NPs themselves have a spherical shape of 10 nm on average. Based on these results and SEM image analysis, we assumed that small spherical Ag NPs are formed in the form of agglomerates/clusters and as the weight percentage of Ag NPs loading increases, the NPs are distributed inside the bulk rather than on the surface of the PLA-NPs film composites. The SEM images of PLA-TiO_2_ film composites presented in Figure 9d,e,f show spherical PLA-TiO_2_ in the form of agglomerates in the PLA surfaces. From these SEM images, it was not possible to distinguish the differences between the PLA-NPs film composites with different NPs loading. The SEM images PLA-ZnO film composites, are shown in Figure 9g,h,i and reveal the presence of spherical and hexagonal nanoparticles agglomerate/clusters, relatively uniformly distributed in PLA film. 

The systematically presented results of PLA-NPs film composites incorporated with Ag, TiO_2,_ and ZnO NPs obtained from SEM analysis show the same trend of distribution of NPs in PLA matrix film composites. In summary, the morphology of the films with PLA and NPs loaded samples with the smallest amount of NPs loading (Ag, TiO_2_, ZnO) shows that the NPs were distributed in the PLA matrix, as evident in the corresponding EDX mapping analysis. These SEM-EDX mapping of all PLA-NPs show an average distribution throughout the near surface region as well as the deeper of the bulk itself. All NPs are incorporated in the PLA material with the tendency of agglomeration with no ordered distribution. The dependence of NPs loading on the final orientation of the PLA-NPs film, analyzed with an average area of counts plotted for Ag NPs, TiO_2_ NPs, and ZnO NPs, illustrates the preferential orientation of the NPs in the near-surface region of the PLA-NPs film composites. 

In order to further understand the surface properties of PLA-NPs films composites in more details, analysis of roughness, and surface free energy measurement were followed. 

#### 2.2.2. Roughness Analysis

**Figure 10:**
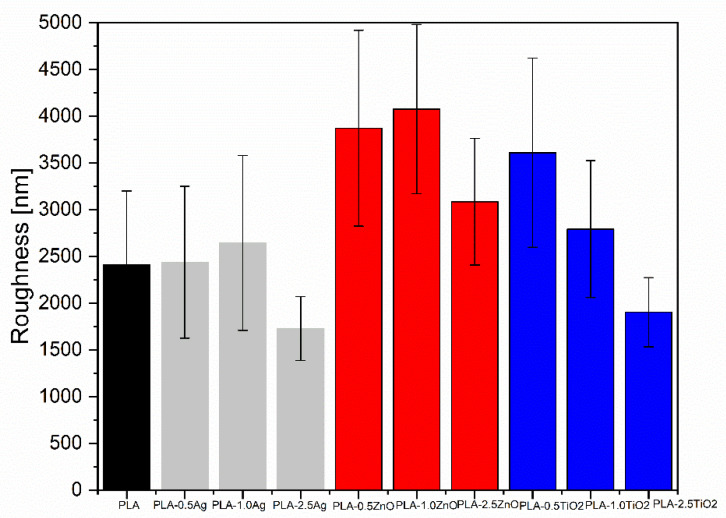
Values of average roughness of the PLA-NPs composites film contain Ag NPs, TiO_2_ NPs, and ZnO NPs.

In Figure 10, the average roughness of the PLA-NPs composite film was shown. The value of roughness varies between PLA-NPs composite films and depends on the NPs weight fraction. All PLA-NPs composite films with max % added NPs showed the lowest roughness and in the case of PLA-Ag NPs and PLA-TiO_2_ composite films, the roughness is even lower than that of the neat PLA material. More specifically, the composites with PLA-Ag show similar values of roughness as the neat material for 0.5 and 1.0% of NPs addition, but the roughness drops to about 1700 nm at higher concentration. In the case of TiO_2_-based PLA-NPs film, the roughness decreases steadily from about 3600 to 1900 nm with increasing NPs content.

The highest roughness corresponds to the composite with ZnO-based PLA-NPs composite as additive with 0.5 and 1.0% concentration of the added additive mass. It reaches 4000 nm on average compared to 2400 nm of the neat material. 

**Figure 11:**
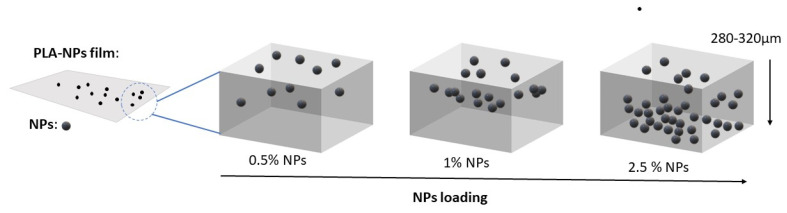
Scheme of possible distribution of NPs in PLA-NPs film composites. With increasing NPs loading the distribution in the inner (bulk) region of material is more or less pronounced.

In general, the roughness behavior of the PLA-NPs composite film explains the previous prediction measured by SEM-EDX mapping analysis regarding the distribution of NPs in different depth layers of the PLA-NPs composite film. The increased roughness of the PLA-NPs composite film at minimum NPs loading outweighs the distribution of NPs at the surface of the PLA-NPs composite film, on the other hand, the distribution of NPs is more or less pronounced with increasing NPs content in the inner region of the PLA-NPs composite film (Figure 11) and thus has no significant influence on the surface roughness changes. Obviously, a different effect on the roughness is also expected due to the different sizes of NPs as well as the agglomeration effect. Among them, the Ag NPs and TiO_2_ NPs with the smallest average size measured with TEM and calculated with XRD (Chapter 2.1) show the least effect on the roughness. In contrast, the ZnO NPs with the largest size of NPs have the largest effect on roughness.

#### 2.2.3. Surface Free Energy Calculations

**Figure 12:**
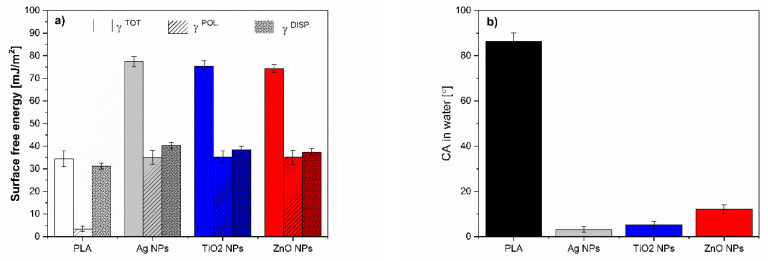
Surface free energy calculation of (**a**) NPs: Ag NPs, TiO_2_ NPs, and ZnO NPs and (**b**) water CA.

**Figure 13:**
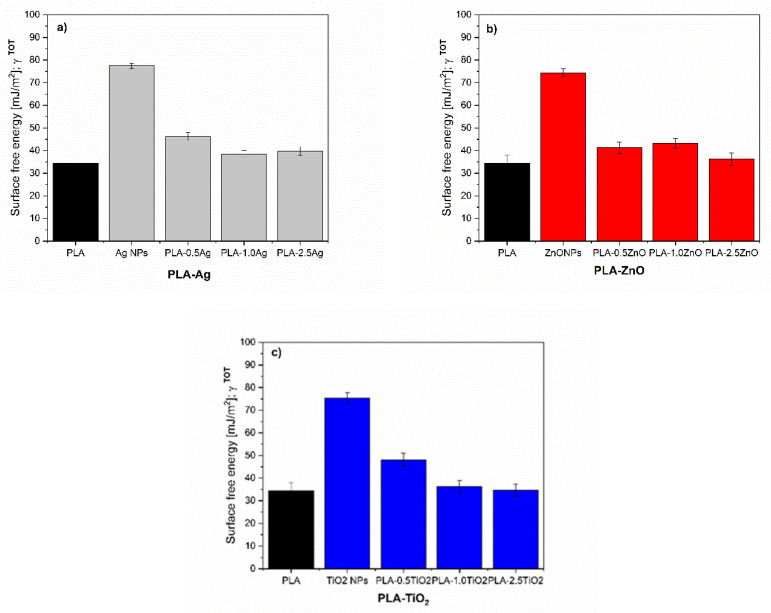
Surface free energy values of neat PLA, and (**a**) PLA-Ag, (**b**) PLA-TiO_2_, and (**c**) PLA-ZnO films composites.

The surface free energy of the reference neat PLA and PLA-NPs composites film was calculated using the Owens and Wendt approach and is presented in Figure 12 and Figure 13, and Table 1 respectively.

The NPs: Ag NPs, TiO_2_ NPs, and ZnO NPs with γ_S TOT_ about 75–80 mJ/m^2^ are known as materials with high surface free energy, where the polar and dispersive fractions are almost equal (Figure 12a). In metals, the surface free energy is high due to the strong metallic bonds between the metal atoms. The NH_2_ groups in Ag NPs, COOH groups in TiO_2_ NPs, and hydroxyl groups in ZnO NPs introduce polar components and contribute to the polarity of NPs surfaces, as also shown by water CA (Figure 12b). Due to the higher surface energy of NPs compared to the surface tension of water (72 mN/m), they tend to minimize the free energy to be wetted by water. On the other hand, the small increase of dispersive components of NPs could be explained by the interactions including van der Waals forces such as London dispersion force, Debye induction force, and Keesom orientation force. The average contact angle of water was much below 90° for all NPs, indicating that all NPs fillers themselves have a hydrophilic nature. 

The results in Figure 13 show the total surface energy of PLA and all the fabricated PLA-NPs composite film. In addition, the total surface energy was calculated, and the dispersive and polar fractions were determined. The solid surface energy of neat PLA is 34.24 mJ/m^2^, which is divided into two main components; the dispersive component has a relatively high value of 31.5 mJ/m^2^, indicating the dispersive interactions including van der Waals forces, while the polar component is low and is 3.4 mJ/m^2^. It was found that the addition of NPs had an effect on the change of total surface free energy compared to the reference samples, neat PLA. An overall increase in SFE was observed for PLA-0.5Ag (i.e., 33%), Table 1. The bonds in polymeric materials are typically weak (Van der Waals), resulting in low SFE of the polymers. When the metals were strongly bonded into the polymer network with a high degree of order at the surface, the surface free energy usually increases. Thus, the increase of SFE in PLA-0.5Ag shows somehow that the metals interact with the polymer chains, with a small increase in the polar fraction, which may indicate the interaction in the region of hydrogen bonding and dipole-dipole interactions and the presence of NPs with polar NH_2_ groups on the surface.

With an increase in Ag NPs concentration (at 1.0%) (Figure 13a) added to the PLA, the total amount of SFE decreased with a decrease in the major dispersion fraction compared with neat PLA. This was somehow expected due to the fact that Ag NPs additives have a basic character (Figure 2) and thus with their incorporation into the surface layer the polar character is more pronounced. The polar interactions are known to be divided into acid and base components [74,75]. At even higher addition of Ag NPs in PLA polymer (PLA-2.5Ag, Figure 12a), the total amount of SFE decreases compared to the sample of lowest addition (PLA-0.5Ag) but approaches the similar values of the polar fraction. The decrease in surface free energy could be due to the distribution of NPs in the deeper layer of PLA-Ag film composites, resulting in lower surface efficiency of them and decrease in surface energy compared to PLA-0.5Ag, where obviously better and more ordered interactions of the nanofiller with PLA are achieved. 

The higher surface energy of all samples with incorporated Ag NPs (PLA-0.5Ag, PLA-1.0Ag, and PLA-2.5Ag) compared to neat PLA clearly shows the integration of NPs on the PLA-composite surface, while NPs has almost twice the polar part of neat PLA. Small changes and indistinct differences between polar and non-polar fractions may also be due to the heterogeneity of the material and the poor NPs distribution in the polylactide surfaces, which is mostly in disordered form (agglomerates were formed as shown by SEM) and not strongly bound or strictly oriented in the material. 

A very similar trend for PLA samples as a function of the concentration of added NPs is observed for PLA-ZnO (Figure 13b). At the lowest concentration of added ZnO NPs (0.5-ZnO), the 18% increase in total SFE is observed, while at higher addition of ZnO NPs (1.0-ZnO NPs), the total SFE is slightly decreased. A 2.5% increase in SFE is observed for PLA-1.0ZnO over neat PLA, while only a 5% increase is observed for PLA-2.5ZnO. With increasing concentration of added ZnO NPs (from 0.5 to 2.5%), the basic polar components decrease negligibly, dominating the dispersive energy fraction. The latter could indicate poorer availability of NPs on the surface or its poor dispersion and interaction state with PLA. Obviously, no well-defined network was performed, but disordered and agglomerated particles were randomly arranged in matrices with non-polar and dispersive interactions. However, a small increase in the polar fraction compared to neat PLA can be attributed to the polar character of ZnO NPs, especially due to the presence of polar hydroxyl groups.

For the PLA samples with addition of TiO_2_ NPs, the highest increases in overall SFE are observed for the smallest mass addition of these NPs compared to the reference sample with the general trend of increasing their polar fraction as well as the non-polar fractions (Figure 13c). In the presence of these NPs on the surface, the increase in the polar fraction is expected due to the availability of polar carboxyl groups on the surface. The decrease in surface free energy for the other two samples, i.e., PLA-1.0TiO_2_ and PLA-2.5TiO_2_, indicates that the TiO_2_ NPs are less accessible and/or more disordered in the PLA matrix at the highest concentrations on the surface of PLA-TiO_2_. This is particularly pronounced for PLA2.5-TiO_2_. However, again, due to the increase in polar fractions in these samples compared to neat PLA, it is suspected that some amount of TiO_2_ NPs with polar carboxyl groups is present on the surface which also proved with a bit better water wettability as for neat PLA.

**Figure 14:**
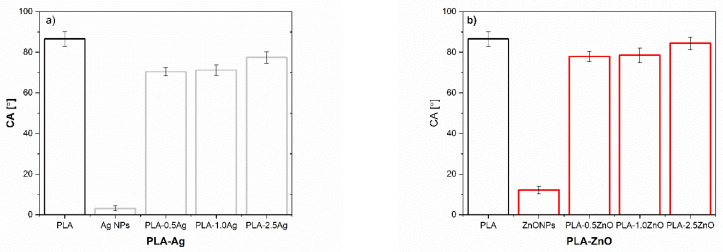

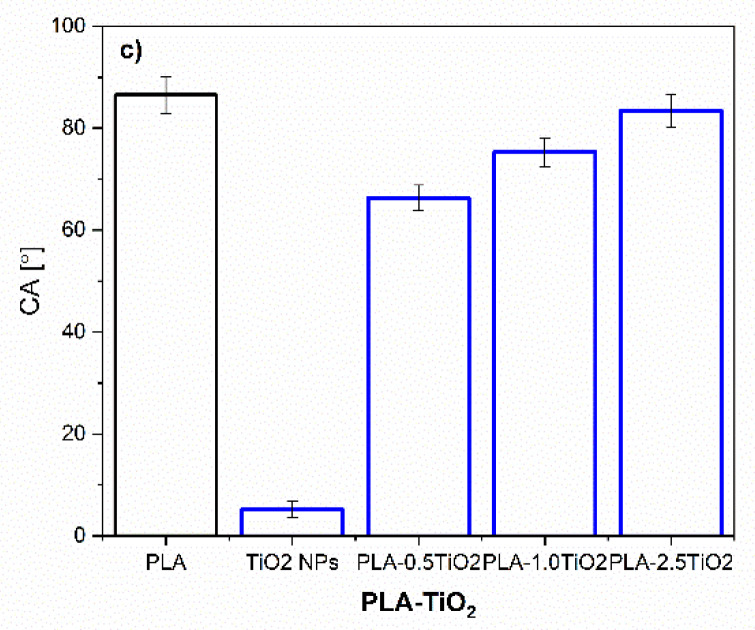
The wettability of neat PLA and (**a**) PLA-Ag, (**b**) PLA-ZnO, and (**c**) PLA-TiO_2_ film composite.

Figure 14 represents the wettability of PLA and PLA-NPs in water respectively. The average contact angle in water was less than 90° for all PLA-NPs composite films, indicating that all of these materials have a hydrophilic nature. For all samples, regardless of neat PLA, an increase in teh polar part is monitored, as evidence by the decreased of the water contact angle. This could be because the introduction of polar groups onto the surface also suggests that the interaction between NPs and PLA is to some extent based on hydrogen bonding which was discussed in our previous publication [36].

Since the dispersive fractions of SFE dominate in all PLA-NPs films composites, these may also recommend a disordered distribution of NPs in the PLA matrix surfaces. The difference in SFE of PLA-NPs can be connected to the preferential distribution of NPs at lowest content of NPs at the surface of PLA-NPs film composites. At an amount of added 0.5% NPs, the surface free energy increases slightly, while it, in general, decreases with larger addition of NPs, which may be related to the mass and mainly due to changes in the morphology structure of the surface of PLA-NPs.

**Figure 15:**
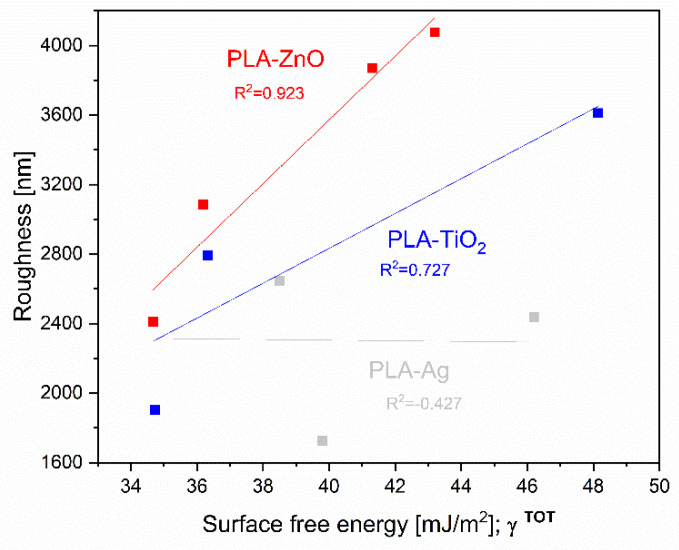
Correlation between roughness and total surface energy of PLA-NPs film composites.

The latter may also be supported by Figure 15 where correlation among roughness and surface free energy is given and may suggest some dependency of among both surface parameters for specific samples. It can be concluded that increased roughness (with decreased amount of NPs) in general increases the total surface free energy. This is in accordance with the calculated linear correlation coefficient that measures the strength and direction of the linear relationship between two variables, roughness and surface free energy, most pronounced for PLA-ZnO and PLA-TiO_2_ and not for PLA-Ag.

#### 2.2.4. Functional Properties of PLA-NPs Films Composites

While surface free energy measurements and roughness study of PLA-NPs films composites verified the presence of antimicrobial NPs at the surface, preferentially at the low wt.% loading NPs, SEM analysis with measurements of elemental mapping allowed the determination of the distribution of NPs as well at the surface region as in the PLA matrix itself. Therefore, the knowledge of mechanical properties of the PLA-NPs composites and antibacterial efficiency with the bacteria adhesion of the potentially used PLA-NPs film in the food packaging and medical devices are unavoidable [6,18,76].

**Antibacterial (Functional) Efficiency of PLA-NPs Films**

**Figure 16:**
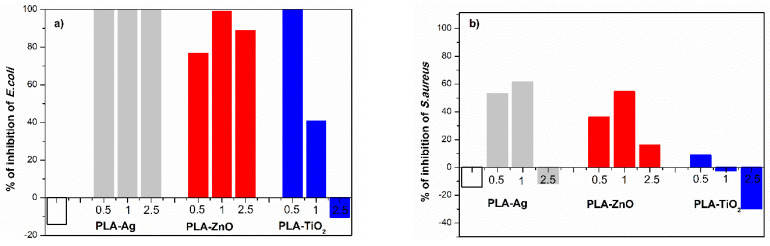
Antimicrobial effect of PLA and PLA-NPs films composites containing Ag NPs(PLA-Ag), ZnO (PLA-ZnO) and TiO_2_ NPs(PLA-TiO_2_) with 0.5, 1.0 and 2.5 wt% of NPs. Percentage of inhibition of (**a**) *E.coli* and (**b**) *S.aureus* were evaluated after 6 h of incubation of bacteria.

Figure 16 summarizes the antimicrobial activity of *E.coli* and *S.aureus* on PLA and PLA-NPs composite films after 6 h incubation time and when three different concentrations of NPs (0.5%, 1.0%, and 2.5%) were added into PLA.

The significant differences in the inhibition of *E.coli* between PLA and PLA-NPs composite films were shown. In the case of neat PLA film, it was clear that PLA exhibited lower antimicrobial activity after 6 h of incubation than in the case of PLA-Ag, PLA-ZnO, and PLA-TiO_2_ composite films. The Ag NPs incorporated into PLA matrix, regardless of the mass of nano-additive to PLA, exceeded the antibacterial activity (100%) after 6 h.

The films containing 0.5 wt% TiO_2_ NPs in PLA showed very high antimicrobial activity of PLA-0.5TiO_2_ after 6 h with 100% inhibition of *E.coli.* With increasing NPs loading, PLA-1.0TiO_2_ film composites also reached 50% of antimicrobial activities, but the film with the highest amount of adequate NPs, 2.5 wt%, showed the same antimicrobial activities as neat PLA, i.e., indicating no antimicrobial activity (even stimulating growth of bacteria). The efficiency of antimicrobial activity of PLA-ZnO films varied and with increasing loading of PLA matrix with ZnO NPs, the percentage of inhibition increases and after 6 h PLA-1ZnO reached 100% of inhibition of *E.coli* (Figure 16a), whilst those with the addition of 2.5% ZnO around 90%.

Summarizing, the results of antibacterial activity against PLA, PLA-NPs films show that the Ag, ZnO, and TiO_2_ NPs provide the antibacterial activity in the PLA-NPs composites, but the strongest antimicrobial activity is observed in the case of PLA-Ag films.

**Figure 17:**
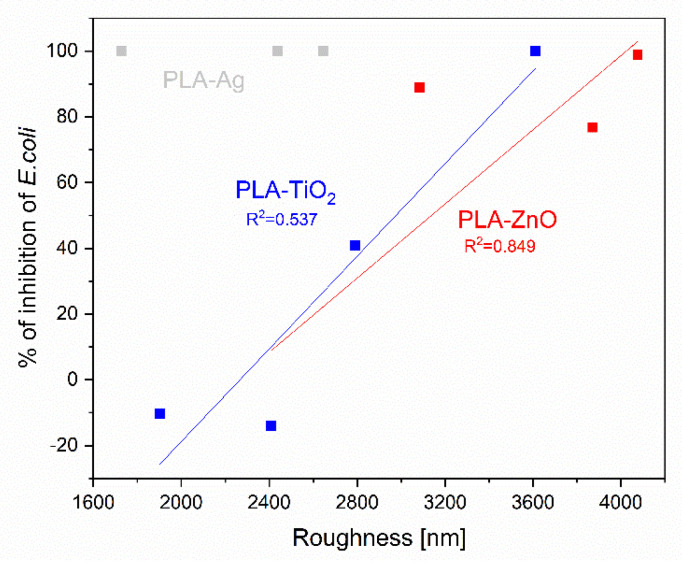
Correlation between inhibition of *E.coli* and roughness of PLA-NPs film composites.

Considering that the availability of NPs on the surface increases with decreasing NPs concentration, it cannot be seen a clear relationship between the NPs concentration on the surface and the antimicrobial efficacy. Obviously, not only the presence of active filler but also the activation form is crucial for the antimicrobial activity: i.e., in most cases, the positively charged metal possesses the antimicrobial activity, which may not always be present in this form in such composites [77]. As already indicated, structural reorganizations occur when NPs are integrated into PLA matrices [36], which consequently altered the surface properties. Therefore, it is important to understand the influence of specific surface parameters on antimicrobial activity. It seems that on the inhibition of *E. coli,* roughness has some influence (Figure 17), which can be seen only for the samples PLA-TiO_2_ and for PLA-ZnO (Figure 17) with some relationship between the two variables. The rough surface obviously has a larger surface area with more hydrophilic character, and it is thus more bacteria inhibiting efficient. No strength of the relationship between the PLA-Ag and roughness is seen [78].

Our continuous work of antibacterial properties of PLA-NPs films was subjected to an antibacterial test which also focused on *S.aureus* (Figure 16b). Considering the main parameters affecting the antibacterial activities of PLA-NPs films, the inhibition of bacterial growth of PLA-NPs films was shown in Figure 16b.

As in the study of antimicrobial activity of *E.coli*, in this work efficiency measurement was also performed on PLA-NPs films based on Ag, ZnO, and TiO_2_ NPs and were measured after 6 h incubation of *S.aureus* in agar. The percentage of inhibition of *S.aureus* increases in the PLA film composites based on Ag NPs and ZnO NPs in the polymer (Figure 16b), but as in the previous case, the films studied with *E.coli* do not reach the maximum value of inhibition of *S.aureus* (Figure 16b).

The antibacterial activity results also show that Ag NPs and ZnO NPs are responsible for some activity against *S. aureus* in the PLA-NPs composites, in contrast, TiO_2_ in the PLA matrix does not accelerate the antibacterial activity for these gram-positive bacteria. It is seen that some antimicrobial activity is observed in the PLA-NPs films with 1.0 wt% and 0.5 wt% NPs loading. Therefore, a 50% inhibition is observed for the PLA-0.5Ag NPs sample and a small increase to 60% for PLA-1Ag. A similar trend is observed for PLA-0.5ZnO and PLA-1ZnO where the inhibition increases from 35% to 55%. At the highest addition of NPs by both types of samples, PLA-2.5Ag and PLA-2.5ZnO the reduction is the least and by sample PLA-2.5 ZnO NPs the growth of bacteria is stimulated. The PLA-TiO_2_ film with the lowest content of NPs, i.e., 0.5% reached 10% of inhibition whilst the other two PLA-TiO_2_ films are not effective.

In general, the presented results show that PLA-NPs films based on Ag and ZnO NPs accelerate the antibacterial activity on *E.coli* rather than *S.aureus*. *Escherichia coli* and other coliform bacteria are important and very present foodborne pathogens also in packaging systems, especially in meat products, cereal products, and vegetables, so it is a great success to be inhibited by developing an active approach.

It appears that the antimicrobial activity of PLA-NPs films largely depends on the cell wall structure of bacteria and the surface structure (modification) of PLA-NPs composite films and the percentage of loading NPs to PLA. Gram-positive and Gram-negative bacteria excelled two main differences in their structure; the cell wall of Gram-negative cell walls are more complex such as *E.coli* have a thin peptidoglycan layer with an outer membrane of lipopolysaccharides which increase the negative charge. On the other hand, the Gram-positive bacteria like *S.aureus,* contains a thicker peptidoglycan layer, in the cell wall with positive charge [14,53]. These results are in agreement with results published in previous work showing that Ag ZnO and TiO_2_ NPs cause antibacterial properties but lower antibacterial activity on *S.aureus* than on *E.coli.* On the other hand, PLA metal-based NPs films, prepared by commonly used methods such as solution processing, solvent casting, electrospinning, showed similar antibacterial activity against *E.coli* and *S.aureus* [44,46,58,79,80,81] as NPs themselves.

It must be considered that all these NPs are also stabilized with the introduction of functional groups (Figure 2), which may have some effect on microbial inhibition. For example, the aminopropylethoxysilane coated metal-based NPs have also been reported to exhibit antibacterial properties [48]. In addition, the morphological properties of Ag NPs also have a significant effect on their antimicrobial properties and activity.

**Figure 18:**
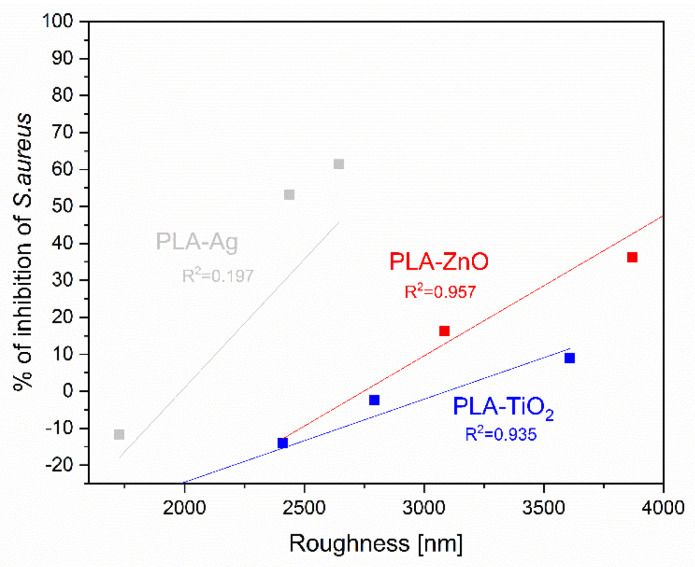
Correlation between inhibition of *S.aureus* and roughness of PLA-NPs film composites.

It has been shown that there is a specific correlation between surface roughness and antimicrobial activity for *E. Coli* (Figure 17) and even more pronounced (for a wider range of samples) is that of *S. Aureus* (Figure 18). Moreover, smaller particles tend to be more active with similar surface ligands [82] as also shown in our work.

Material surface properties, including high surface roughness, higher surface free energy, and lower contact angle compared to neat PLA, can apparently play a key role in enhancing the antibacterial behavior of surfaces for all our composites, which has also been demonstrated previously for plastics materials sprayed with copper and silver [78]. It can be concluded that surface accessible amount of filler (NPs) does not directly and linearly influence final antimicrobial activity. However, integration of nanoparticles leads to surface physicochemical and morphological properties that enhanced/altered the final material antimicrobial activity.

In general, understanding the antibacterial effect of functional polymer surfaces such as PLA metal-based NPs composites films is a complex topic and not yet known in detail. Nevertheless, it can be concluded that not only the chemical composition causes the functional response, but also the free energy state and the morphology of a surface may change the microbial inhibition.

**The Surface Mechanical Properties of PLA-NPs Film**

The surface mechanical properties of PLA NPs are largely determined by the interaction between the NPs and PLA, crystalline morphology, and structure. In previous studies, PLA was shown to interact (adsorb) with NPs and not provide additional sites for nucleation. Considering the rather acidic behavior of TiO_2_ NPs and ZnO NPs and the presence of carboxyl and hydroxyl groups on the surface compared to Ag NPs, the hydrogen bonding between NPs and PLA is present [36]. In this section, we aim to clarify the influence of added NPs on the mechanical properties of PLA-NPs film composites. Nanoindentation tests can provide important information on the near-surface mechanical properties and deformation behavior of solids. Submicron measurements of material properties are important not only for characterizing increasingly small electronic and mechanical devices but also for analyzing thin interfacial films that control friction and adhesion, which is also important for packaging where two media interact.

**Figure 19:**
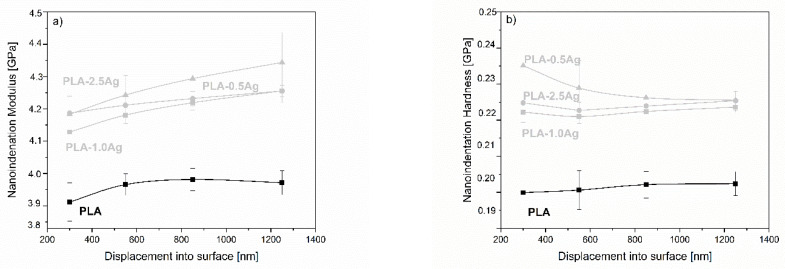

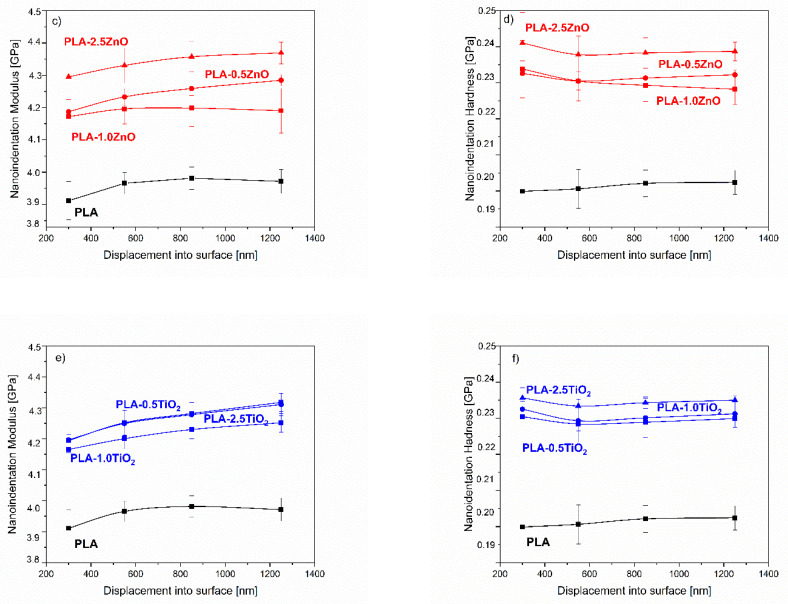
Values of nanoindentation modulus and hardness as a function of penetration depth for materials with different nanofillers. From top to bottom: (**a**,**b**) Ag NP, (**c**,**d**) ZnO NPs, and (**e**,**f**) TiO_2_ NPs.

To further investigate the distribution of NPs in PLA films at different depths, the nanoindentation modulus and hardness were measured up to a maximum depth of 1300 nm. The range was divided into intervals (200–400 nm, 400–700 nm, 700–1000 nm, and 1000–1300 nm), and the nanoindentation modulus and hardness were averaged over each interval. The resulting values as a function of the mean value of the indentation depth for each interval are shown in Figure 19. There is a significant increase in the values of modulus and hardness due to the addition of NPs compared to the neat material. The increase in moduli varies in the range of 0.5% to a maximum of 3.8% for the maximum concentration of Ag NPs compared to the minimum analyzed depth. PLA-NPs composite films with Ag NPs as filler show the steepest increase in modulus with increasing penetration depth. The least change is observed for TiO_2_ NPs, whose modulus can be considered constant over the analyzed penetration depths. Considering the absolute values of modulus, the largest values correspond to the ZnO NPs composite, which has the largest values of modulus among all tested materials at the highest particle concentration, but the changes with penetration depth are insignificant in the range of 1.0%. The results for hardness show the same trend as for modulus; hardness increases with penetration depth compared to neat PLA but decreases slightly with increasing NP loading. It seems that the incorporation of NPs makes the surface of PLA NPs harder, while the effect is less pronounced with increasing NP loading. Please note that the interpretation of nanoindentation results at such shallow depths for relatively soft materials can be very challenging due to surface irregularities and the limitations of the measurement method.

**Figure 20:**
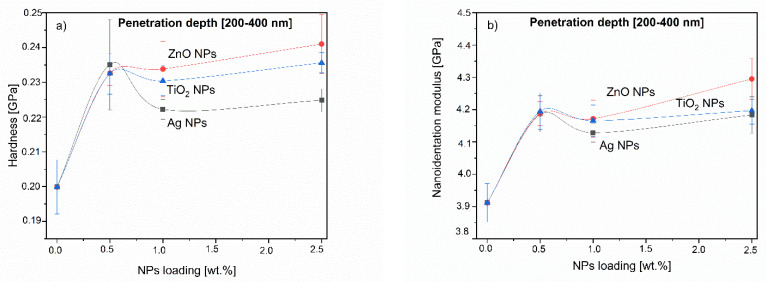
The nanoindentation modulus (**a**) and hardness (**b**) in relation to NPs loading of PLA-NPs composites.

Figure 20 shows the results in the relatively shallow penetration region, where the surface effects and measurement artifacts were no longer expected. It can be clearly seen that the mechanical properties of PLA-NPs depend on the NP concentration and a particular increase is observed at a concentration of 0.5% of NPs addition. This peak is observed for all the analyzed depths. This supports the hypothesis of a near surface distribution of NPs for materials with 0.5% addition. A further increase of the concentration to 1% shows a decrease of the modulus compared to 0.5%. The hardness remains almost stable and even decreases for PLA-1Ag composite. Materials with a maximum concentration of 2.5% additives show an increase in modulus and hardness values compared to the other concentrations, which also supports the hypothesis. As the penetration depth increases, these values become higher and reach the maximum of the observed values, which means that the measurement depth penetrates or approaches the layer of particles distributed deeper in the material by detecting their presence at greater depth (similar to the substrate effect). It is logical that a higher concentration of NPs in deeper layers of the film leads to a higher reinforcement and increase of the measured values from an absolute point of view, but the relative effective increase of the property values caused by 0.5% NPs addition cannot be neglected.

The nanoindentation results clearly showed that the incorporation of NPs in PLA matrix improved the mechanical properties of the PLA-NPs composite film. There are several possible reasons for enhanced mechanical properties of PLA-NPs composites film, one being the addition of the highly oriented crystalline structure of the NPs (previously explained by XRD measurement of the NPs) compared to the semi-crystalline PLA polymer, and the other being the improved interaction of NPs and PLA.

## 3. Materials and Methods

### 3.1. Materials

PLA, poly(*L*-lactic acid), neat, were obtained from Plastika Kritis S.A. (Iraklion, Greece) with molecular weight *M*_w_~75 kg/mol and intrinsic viscosity *η* = 1.24 dL/g. As it was reported in our previous work [36], the NPs additives have been added to PLA matrix with different loading: (i) Ag NPs were purchased by Inframat Advanced materials (Manchester, NH, USA), (ii) ZnO NPs supplied by Sigma Aldrich (Saint Luis, MO, USA) and (iii) titanium oxide (TiO_2_) NPs obtained from Cinkarna Celje, Slovenia (product name CCA 100 BS).

#### Preparation of PLA-NPs Composites Films

All PLA-based metal nanoparticle additives films (PLA-Ag, PLA-ZnO, PLA-TiO_2_) were prepared by melt-mixing methods with different loading of NPs (0.5, 1.0, and 2.5 wt%) as shown in the previous study [36]. PLA granulates and NPs were left overnight in a vacuum oven at 110 °C. The dried materials were prepared in a corotating twin screw melt mixer, with 30 rpm, at 195 °C for 10 min. The Paul-Otto Weber (Germany) thermal press was heated to the temp. around 180 °C. The 2 g of the used PLA-NPs were weighed and put in the press between two steel plates and used a tonnage load applied while the sample is melted and being pressed for 3 min. The thin films (280–320 μm) of PLA-NPs between two plates were followed by a sudden cooling between two ice plates.

A complete overview of the prepared PLA-NPs composites is presented in Table 2, with defined names for each composite’s mixture.

### 3.2. Methods

#### 3.2.1. NPs Characterization

**XRD Measurement**

NPs additive of Ag, ZnO, and TiO_2_ were evaluated to study the crystalline phases and orientation. The XRD spectra were recorded by MiniFlex II XRD system (Rigaku Co., Japan), with Cu Kα radiation (0.154 nm), over the 2θ range from 5° to 50° with scanning rate of 1 deg/min. The average crystallite size was calculated using the well-known Scherrer formula:

Crystallite size[nm] = kλ/βcosθ,(1)

where λ is the X-ray wavelength in nanometer (nm) (0.154 nm for Cu Kα), β is the diffraction peak width at the half maximum height (FHWM) resulting from small crystallite size in radian and k is a constant related to crystallite shape, normally known as 0.9. The average crystallite size was evaluated by fitting Gaussian–Lorentzian curves and using Origin Pro software.

**TEM Analysis**

The NPs were characterized using transmission electron microscopy (TEM, JEM-2100, Jeol Ltd., Tokyo, Japan) operated at 200 kV. For the TEM investigations the NPs were deposited on a copper-grid-supported transparent carbon foil.

**ATR-FTIR Spectra**

To study the chemical structure of the surface functional groups of NPs (Ag, ZnO, and TiO_2_), ATR-FTIR spectra measurements were performed using the Spectrum GX instrument (Perkin Elmer, Waltham, MA, USA) equipped with a diamond crystal. Each spectrum was determined as the average of 32 scans with a resolution of 4 cm^−1^. The spectra of the nanomaterials were measured in the range of (400 to 4000) cm^−1^ at room temperature. All spectra were baseline corrected and smoothed after the measurements.

**Zeta Potential Measurements**

Zetasizer Nano ZS (Malvern Instruments, Worcestershire, UK) equipped with a He-Ne-laser (wavelength of 633 nm) were used for electrokinetic measurements of zeta potential (ZP) at 25 °C. The pH of the Ag, ZnO, and TiO_2_ suspensions was adjusted with HCl (0.1 mol/L) or NaOH (0.1 mol/L).

#### 3.2.2. PLA-NPs Composites Films Characterization

**Morphology and Elemental Distribution Measurements**

The morphology, elemental composition, and elemental surface distribution of neat PLA and PLA-NPs film were examined by a field emission scanning electron microscopy (FE-SEM) using Verios G4HP device (Thermo Fisher Scientific, Waltham, MA, USA) equipped with energy-dispersive X-ray (EDX) spectroscopy (Quanta 650-v9, Thermo Fischer Scientific, Waltham, MA, USA). All PLA-NPs films were sputter coated with conductive layer of carbon (CED Balzer Baltec, Schalksmühle, Germany).

**Surface Roughness Measurements**

For surface roughness measurements the scratch testing was performed with G200 Nanoidenter (Agilent, Santa Clara, CA, USA). Method of scratch testing with topographic compensation was utilized, however, only surface roughness data has been extracted and evaluated. It is obtained by recording displacement of the tip during scanning of the initial sample surface with an applied small force of 20 µN. The scanning was done on the distance of 500 µm and profiling velocity of 10 µm/s. Roughness obtained by this method is measured in nanometers (10 scratches per sample were performed). These measurements were performed at least 200 µm apart.

**Contact Angle Measurements and Surface Free Energy Quantification**

To highlight the phenomena of surface properties of PLA-NPs composite films, they were investigated with contact angle measurements. Contact angle measurements and surface free energy (SFE) calculations of all PLA-NPs composites were measured using a goniometer from DataPhysics (Filderstadt, Germany) and were performed with two different liquids: ultra-pure water (Millipore, Burlington, MA, USA), diiodomethane (Sigma-Aldrich, Burlington, MA, USA 99%) with a droplet volume of 3 μL and an average of at least three liquid droplets per surface. In this way, static contact angles (SCA) were measured at room temperature. The approach of Owens, Wendt, Rabel, and Kaelble (OWRK) represents one of the most common methods for calculating the SFE of polymeric materials, using water and diiodomethane as measuring fluids [61,83,84] and following the well-known Young equation:

γ_s_ = γ_sl_+ γ_1_ cosθ, (2)

where γ_s_ is the SFE of a solid, γ_sl_ is the SFE with respect to the solid-liquid interface, γ_1_ is the SFE of a measuring liquid, and θ is the contact angle between the solid and the measuring liquid. Although Fowkers, who pioneered the idea of dividing the SFE into individual components, followed the prediction that the interfacial interaction depends on the properties of the measuring liquid and the SL of the solid under study, our research followed the Owens, Wendt, Rabel, and Kaelble approach (OWRK) to calculate the SFE, where the interfacial energy (γ_sl_) of PLA-NPs composite films was obtained by summing the surface free energies of the individual phases, divided into their dispersive (D) and polar (P) interphase interactions. The dispersive and polar phases of the surface energy of the PLA-NPs composite films is calculated by using two liquids with known surface tensions such as diiodomethane with a surface tension of 50.8 mN/m, polar component 2.3 mN/m, and dispersive component 48.5 mN/m, and in contrast, water with a surface tension of 72.8 mN/m with polar component 51.0 mN/m, dispersive component 21.8 mN/m as one polar component [83,85].

The surface free energy evaluation of NPs was measured in the form of a pellet. The NPs dispersion was dried overnight at 100 °C in an oven. The dried NPs were ground into fine powder and a small amount of the NPS powder was added to the collar of Perkin Elmer Hydraulische Presse. The NPs powder was pressed for 2 min to form a pellet.

**Antibacterial Properties**

The antimicrobial activity of PLA films and PLA-NPs composite films against the bacteria *Escherichia coli* (DSM 1576) and *Staphylococcus aureus* (DSM 799) was determined according to the internal protocols of the Department of Microbiological Research, Center for Medical Microbiology of the National Laboratory for Health, Environment, and Food in Maribor, No. P96 ‘Biofilm production on various materials *Staphylococcus aureus*’ P90 (ISO22196) for determination of microbiological activity/antimicrobial activity of plastic surfaces, reported in our previous works, and they are standardized methods known for plastic surfaces [86,87]. More specifically, the PLA, PLA-NPs composite films of size 10 mm × 10 mm were exposed to the standardized medium inoculated with *Escherichia coli* and *Staphylococcus aureus* and set at 0.5 on the McFarland scale. The antimicrobial performance of the PLA-NPs films were chosen after 6h. After inoculation of the PLA and PLA-NPs film composites, the viable bacteria were evaluated by the pour plate method (plate counting agar was used). The effect of incorporation of metal oxide NPs into PLA matrix composites was evaluated as reduction of bacterial growth and counting of bacterial number after incubation of neat PLA polymer compared to PLA-NPs composite films.

**Nanoindentation**

Continuous stiffness measurement is a nanoindentation characterization method developed by Oliver and Pharr [87], which utilizes the dynamic loading of the sample material. Based on the dynamic model of the system comprised from the nanoindenter setup and sample together, the methodology allows determination of the material properties throughout the whole indentation process not just during the unloading as standardized method. Therefore, CSM also provides significantly more data compared to standardized method, as the latter delivers one value of modulus or hardness corresponding to the prescribed maximal depth or load during the test.

CSM tests were performed with Nanoindenter G200 equipped with XP head and Berkovich indenter for the depth of 2500 nm, the distance between the indents was set to minimum 100 µm to avoid the interference of the stress fields of neighboring indents. In total 36 indents were done on every sample. The Poisson’s ratio was taken as 0.35 for all materials since this parameter does not have a significant effect on the end result. Elastic nanoindentation modulus and hardness were determined throughout the whole penetration depth and analyzed up to 1200 nm in order to avoid the effect of substrate.

## 4. Conclusions with Discussion

The surface properties of polymer-based NPs composites depend on the NPs filler loading and are closely related to the surface structure, morphology, size, and distribution of NPs in the polymer matrix. Our research focused on the surface properties of PLA-NPs composite films reinforced with Ag, ZnO, and TiO_2_ NPs and fabricated by melt-mix extrusion process. Due to the different surface structures of NPs, size and amount of added Ag, ZnO, and TiO_2_ nanoparticles are expected to have different effects on the surface properties, which indirectly explains the antibacterial and mechanical properties of the PLA-NPs film composite.

In general, the incorporation of a small (0.5 wt%) amount of the NPs into the PLA matrix causes an enhanced change in the surface properties of the PLA-NPs composite. The SEM-EDX mapping of all PLA-NPs shows an average distribution throughout the near surface region as well as in the depth of the bulk itself. More so, spectrum mapping analysis and roughness measurements of PLA loaded NPs (0.5%, 1.0%, and 2.5% NPs) predict the preferential distribution of NPs on the surface of PLA-NPs composites, followed by a small amount of NPs accelerating a more intense effect on surface roughness. All PLA-NPs composite films with max % added NPs showed the lowest roughness, which can be attributed to the different size of NPs as well as the agglomeration effect. With the smallest average size measured by TEM and calculated by XRD, the lowest effects on roughness were evaluated. In contrast, ZnO NPs with the largest size of NPs have the largest impact on roughness.

The preferential distribution of NPs on the surface of PLA-NPs film composites was measured and calculated in the SFE of PLA-NPs. For an addition of 0.5% NPs, the surface free energy slightly increases, while it generally decreases with larger addition of NPs, which may be related to the mass and is mainly due to changes in the morphology structure of the surface of PLA-NPs. For PLA-0.5Ag, an overall increase in SFE was observed (i.e., 33%), with a small increase in the polar fraction, which may indicate the interaction in the region of hydrogen bonding and dipole-dipole interactions, as well as the presence of NPs with polar NH_2_ groups on the surface and basic character. At the lowest concentration of added Zn NPs (0.5-ZnO), an increase of 18% in the total SFE is observed, while at higher addition of ZnO NPs (1.0-ZnO), the total SFE slightly decreases. However, the small increase in the polar fraction compared to neat PLA can be attributed to the polar character of ZnO NPs, especially due to the presence of polar hydroxyl groups. For the PLA samples with the addition of TiO_2_ NPs, the highest increases in total SFE are observed for the smallest mass addition of these NPs compared to the reference sample with the general trend of increasing their polar fraction due to the availability of polar carboxyl groups on the surface as well as the non-polar fractions.

Most of the prepared samples were antimicrobially active against *E. coli* and very less against *S. aureus*. The PLA-NPs films based on Ag NPs, ZnO NPs, and TiO_2_ NPs outperformed the antibacterial activity on *E.coli* rather than on *S.aureus*, with the *E.coli* efficiency being the most enhanced in the case of PLA-Ag films. In general, the antibacterial efficacy of the surface properties of the PLA-NPs composite films, including the high surface roughness, higher surface free energy, and lower contact angle compared to the neat PLA against *E.coli* and *S.aureus* does not directly depend on the surface accessible amount of the filler (NPs). However, the integration of nanoparticles leads to surface physical, chemical, and morphological properties that enhance/modify the antimicrobial activity of the final material.

The incorporation of NPs in PLA not only changes the surface properties of the PLA-NPs film but it has been investigated that the presence of NPs in the PLA matrix of the composite film has an improved effect on the mechanical properties of all PLA-NPs film.

## Figures and Tables

**Table 1:** SFE calculations of PLA and all PLA NPs loaded film composites.

Sample	SFE Total (mJ/m^2^)	Dispersive Part (mJ/m^2^)	Polar Part (mJ/m^2^)
PLA-neat	34.58	31.17	3.41
PLA-0.5Ag	46.21	39.77	6.44
PLA-1.0Ag	38.48	26.81	11.68
PLA-2.5Ag	38.48	33.46	6.4
PLA-0.5TiO_2_	48.13	35.81	5.48
PLA-1.0TiO_2_	36.32	38.64	4.61
PLA-2.5TiO_2_	34.73	31.23	4.99
PLA-0.5ZnO	41.29	40.24	7.89
PLA-1.0ZnO	43.25	27.98	8.34
PLA-2.5ZnO	36.22	29.69	5.04

**Table 2:** Prepared PLA-NPs composites.

Name of Material/NPs Loading (wt.%)	0.5	1.0	2.5
PLA-Ag	PLA-0.5Ag	PLA-1.0Ag	PLA-2.5Ag
PLA-ZnO	PLA-0.5ZnO	PLA-1.0ZnO	PLA-2.5ZnO
PLA-TiO_2_	PLA-0.5TiO_2_	PLA-1.0TiO_2_	PLA-2.5TiO_2_

## Data Availability

Data is contained within the article.

## Sample Availability

Samples of the compounds are available from the authors (K.P.Č).
